# Ultrasound dynamic monitoring of IVCD to guide application of CRRT in patients with renal failure combined with acute heart failure

**DOI:** 10.1038/s41598-022-17375-w

**Published:** 2022-08-18

**Authors:** Guang Yu, Shaoyu Tao, Yingzhi Jin, Wanxia Li, Zanqun Hu, Xiaowei Fang

**Affiliations:** 1 Department of Emergency and Critical Care Medicine, The Second Affiliated Hospital of Nanchang University, No. 1 Minde Road, Nanchang, 330006 Jiangxi China; 2 Department of Clinical Medicine, The Second Clinical Medical College, Nanchang University, Nanchang, China

**Keywords:** Medical research, Renal replacement therapy

## Abstract

We explored the application value of bedside ultrasound dynamic monitoring of the inferior vena cava diameter (IVCD) and collapse with sniff (inferior vena cava collapsibility index [IVCCI]) to guide dehydration adjustment in continuous renal replacement therapy (CRRT) in patients with combined renal failure and acute heart failure. We selected 90 patients with combined renal and acute heart failure who required CRRT in the intensive care unit (ICU) from January 2019 to June 2021. According to different blood volume assessment methods, patients were randomly divided into ultrasound, experience, and control groups. We compared serum creatinine, potassium, and N-terminal pro-brain natriuretic peptide (NT-proBNP) levels; time to improved heart failure symptoms; CRRT time; ventilator use; ICU length of stay; vasopressor use; and incidence of adverse events among groups. There were no significant differences in serum creatinine, potassium, and NT-proBNP levels in pairwise comparisons among groups before and after CRRT (P > 0.05). The time to improved heart failure symptoms, CRRT time, and ICU length of stay in the ultrasound and experience groups were lower than those in the control group; the differences were statistically significant (P < 0.05). Ventilator use duration was lower in the ultrasound and experience groups compared with the control group, with a statistically significant difference between the ultrasound and control groups (P < 0.05). The duration of vasopressor use in the ultrasound and control groups was lower than that in the experience group; the difference was statistically significant (P < 0.05). The incidence of adverse events was lower in the ultrasound group compared with the experience and control groups; the difference was statistically significant (P < 0.05). Ultrasound dynamic monitoring of IVCD and collapse with sniff can accurately assess blood volume status, and provide guidance for dehydration adjustments in CRRT and rapid relief of heart failure symptoms in patients with combined renal and acute heart failure.

## Introduction

Renal failure combined with acute heart failure is a clinical emergency and critical illness characterised by rapid disease progression, long hospital stays, and high mortality rates, which seriously threaten patient safety^1^. In clinical practice, the main treatment strategy is to relieve the heart failure symptoms, including strengthening the heart, diuresis, and vasodilation^2^. However, owing to renal failure, the accumulated metabolites and blood volume in these patients cannot be excreted through the kidneys. Hypertension and overload are generally poorly treated with only routine diuretic and vasodilator therapy, whereas continuous renal replacement therapy (CRRT) can restore kidney damage with extracorporeal circulation blood purification that continuously removes metabolites and excess blood volume in the body, thereby reducing pre- and post-cardiac load and effectively improving the symptoms and overall condition of patients with heart failure^3^.

However, various complications frequently occur during the clinical application of CRRT; hypotension is one of the main complications^4,5^. Some studies have shown that the degree of blood volume decline is an important cause of blood pressure changes during CRRT. Excessive and rapid dehydration exceeds tissue fluid reflux, leading to insufficient effective blood volume and hypotension^6^. Correctly assessing the patient’s blood volume status during CRRT and formulating an optimal dehydration protocol is a difficult problem faced by clinicians.

Recently, ultrasound monitoring of the inferior vena cava diameter (IVCD) and variability (IVCD and collapse with sniff, inferior vena cava collapsibility index [IVCCI]) have been widely used in blood volume assessment because of their intuitive, accurate, non-invasive, and repeatable advantages. Previous studies have recommended using the IVCD as a guide for evaluating patients’ blood volume status^7–9^; however, there are few reports on CRRT application in patients with renal failure combined with acute heart failure. Therefore, we aimed to explore the clinical application of bedside ultrasound dynamic monitoring of the IVCD and IVCCI in guiding dehydration adjustment during CRRT in patients with renal failure combined with acute heart failure.

## Materials and methods

This study followed a prospective, randomised controlled design, and was approved by the Biomedical Research Ethics Committee of the Second Affiliated Hospital of Nanchang University. The study was conducted in accordance with the relevant guidelines and regulations. All patients were informed of the potential benefits and risks. Written Informed consent was obtained from all patients.

We selected 90 patients with renal failure combined with acute heart failure admitted to the intensive care unit (ICU) at our hospital from January 2019 to June 2021 who required CRRT. ﻿The mean participant age was 68.23 ± 11.41 years, with 28 women and 62 men.

We included patients who: (1) were age ≥ 18 and ≤ 80 years old; (2) agreed to CRRT; and (3) met the diagnostic criteria for renal failure combined with acute heart failure, according to the Kidney Disease Improving Global Outcomes guidelines for the primary diagnosis and treatment of acute heart failure (2019).

We excluded patients with any of the following: (1) history of malignant tumours or psychiatric diseases; (2) history of congenital heart disease, hypertrophic cardiomyopathy, or pulmonary hypertension; (3) abnormal coagulation function, intracerebral, visceral, or gastrointestinal haemorrhage in the past 3 months, or heparin anti-coagulant contraindications; (4) CRRT time ≤ 12 h; (5) missing data because the inferior vena cava could not be detected on ultrasound; and (6) cardiogenic shock or cardiac ejection fraction ≤ 50%.

The patients were randomly divided into three groups using the random number table method (ultrasound, experience, and control groups). Each group comprised 30 patients. Differences in sex, age, and Acute Physiology and Chronic Health Evaluation II scores were not statistically significant among the three groups, and the participant characteristics among groups were comparable at baseline (Table 1).

**Table 1:** Characteristics of the study population.

Group	Cases	M/F	Age	APACHE II
Ultrasound	30	21/9	67.57 ± 12.97	20.17 ± 6.87
Experience	29	21/8	69.59 ± 10.60	19.28 ± 5.10
Control	29	18/11	68.45 ± 10.64	19.17 ± 4.05
*χ*^*2*^*/F*		0.79	0.23	0.29
*P*		0.68	0.80	0.75

One patient in the experience group had gastrointestinal bleeding, and one patient in the control group discontinued treatment. Two patients were excluded from the statistical analysis.

## Intervention

### Ultrasound group

At the initiation of CRRT, physicians placed patients in a supine position and exposed the chest and abdomen. Then, the 3.5-MHz convex array probe of the Mindray M7 portable colour Doppler ultrasound device was used to measure the section from the IVCD to xiphoid process. Multiple breathing cycles were recorded at a distance of 2.0 cm from the right heart of the inferior vena cava, using M-mode ultrasound. The maximum end-inspiratory diameter (IVCDmax) and minimum end-expiratory diameter (IVCDmin) were measured simultaneously. The IVCD was defined as the IVCDmax, and the IVCCI was calculated according to the following formula: (IVCDmax − IVCDmin)/IVCDmax × 100%. All examinations were completed by the ultrasonography team comprised of physicians with ultrasound qualifications. All physicians were uniformly trained for quality control to ensure rigorous ultrasound data collection. Based on the IVCD measured by the chief ultrasound physician as the conventional true value, the pre-experiment analysis indicated a relative error for IVCD measurements by different physicians of < 0.05 and a relative error of IVCD measurements by the same physician at different time periods of < 0.02. The measurement time for each ultrasound method was approximately 10 to 15 min. Each index was measured three times and the average value was calculated. Physicians adjusted the volume of dehydration according to the IVCD and IVCCI and repeated the above procedure every 4 h until CRRT discontinuation.

Blood volume status was evaluated according to the practical guidelines of the British Society of Echocardiography^10^: IVCD ≤ 2.1 cm with IVCCI > 50%, defined as a low volume status; IVCD ≤ 2.1 cm with IVCCI < 50% or IVCD > 2.1 cm with IVCCI > 50%, defined as a balanced volume status; and IVCD > 2.1 cm with IVCCI < 50%, defined as a high volume status. The daily urine output of healthy people is 1500 ml-2000 ml. For the convenience of calculation, we defined normal daily urine output as 1800 ml with an average urine output of 300 ml every 4 h. Previous preliminary experiments have demonstrated that if the dehydration volume within 4 h exceeded four times that of normal urine output in a high-volume state, the incidence of complications increased significantly; if it exceeded two times that of normal urine output, the time to heart failure symptom improvement and the incidence of complications increased significantly. In a state of volume balance, the incidence of complications increased significantly when the dehydration volume exceeded two times the normal urine volume within 4 h, and the time to heart failure symptom improvement was significantly increased when the dehydration volume was the same as the normal urine volume. The target dehydration volume within 4 h was set as 1000 ml for patients in a high-volume state and 500 ml for patients in a balanced-blood volume state. Since the low-volume state leads to hypotension if dehydration were continued, and rehydration further aggravates heart failure symptoms, physicians adjusted the 4-h target dehydration volume to 0 ml for patients with low volume status (CRRT dehydration volume within 4 h = 4-h target dehydration volume + intake volume within 4 h − urine volume within 4 h).

### Experience group

Physicians adjusted the dehydration amount according to heart rate, mean arterial pressure, central venous pressure, and pulmonary moist rales after CRRT using a general empirical scoring method (Table 2).

**Table 2:** General empirical scoring.

	Score			
	3	2	1	0
HR	≥ 180	140–179	110–139	50–109
MAP	≥ 160	130–159	110–129	65–109
CVP	≥ 16	12.1–15.9	5–12	2.5–4.9
PMR		> 50% of both lung fields	< 50% of both lung fields	Not observed

*HR* heart rate, *MAP* mean arterial pressure, *CVP* central venous pressure, *PMR* pulmonary moist rales.

Scoring was performed every 4 h from the beginning of CRRT to the time the patient was assisted off the machine. Physicians adjusted the 4-h target dehydration volume to 1000 ml, 500 ml, and 0 ml for scores of 8–11, 4–7, and 0–3 points, respectively (CRRT dehydration volume within 4 h = 4-h target dehydration volume + intake volume within 4 h − urine volume within 4 h).

### Control group

From the beginning to discontinuation of CRRT, the target dehydration volume was constant at 100 ml/h, and no volume evaluation adjustments were performed during the treatment process (CRRT dehydration volume within 4 h = 4-h target dehydration volume + intake volume within 4 h − urine volume within 4 h).

### Homogeneous management

In addition to the aforementioned experimental target dehydration adjustment measures, homogeneous management was adopted for all three groups of patients, including primary disease treatment, anti-infection protocol, airway management, mechanically assisted ventilation strategy, maintenance of fluid volume and electrolyte balance (4.0 mmol/l < potassium < 5.3 mmol/l), drug therapy, administration of colloidal fluid supplement such as albumin (to maintain albumin > 3.5 g/l), and nutritional support.

The three groups of patients were treated with the same blood purification machine (PrismaFlex system) and the same mode of CRRT (CVVHD mode). All patients were treated with extracorporeal heparin for local anti-coagulation and protamine neutralisation. Physicians adjusted the dosage of heparin and protamine according to four coagulation parameters (activated partial thromboplastin time maintained within the normal range of 1–1.5 times). During CRRT, blood flow was maintained at 150–200 ml/min and dialysate flow at 2000 ml/h (dialysate formula: normal saline, 2000 ml; sterile volume for injection, 1000 ml; 50% glucose solution, 10 ml; 10% normal saline, 20 ml; magnesium sulphate, 2.5 ml; 10% potassium chloride, 7.5 ml; sodium bicarbonate, 45 ml; and peripherally pumped calcium chloride, 10 ml/h).

When the patient presented with hypotension, dehydration was immediately suspended, and intravenous fluids and vasopressors were administered if necessary (including norepinephrine and dopamine) to maintain the patient’s mean arterial pressure above 65 mmHg.

### Observation indicators

Serum creatinine, potassium, and N-terminal pro-brain natriuretic peptide (NT-proBNP) levels were measured before and after CRRT for 24 h. Data on the time to improved heart failure, CRRT time, ventilator use duration, length of ICU stay, duration of vasopressor use, and incidence of adverse events (including hypotension, arrhythmia, and delirium, but not malignant arrhythmia) were collected during hospitalisation in the ICU. The incidence of adverse events was calculated based on whether the enrolled patients experienced an adverse event.

### Clinical improvement of heart failure

1. Symptom improvement: symptoms were considered improved if chest tightness and dyspnoea were improved by one grade according to the New York Heart Function Classification and the frequency of coughing pink frothy sputum decreased by 20% compared with the previous assessment (endotracheal intubation patients were excluded).
2. Physical signs of improvement: the area of pulmonary moist rales decreased by 50%.

(3) Improvement in monitoring indicators: the heart rate, respiratory rate, central venous pressure, or mean arterial pressure decreased by 20%.

Physicians performed evaluations once per hour, and when patients met the above three conditions, their heart failure was considered improved.

### Statistical methods

Statistical analyses were performed using SPSS 22.0 software (IBM Corp., Armonk, NY, USA). Continuous data are presented as the mean ± standard deviation. Categorical data are described as frequencies and percentages. Differences between the two groups were assessed using Student’s *t*-test for continuous variables or the chi-square test for categorical variables. Statistical significance was set at P < 0.05.

## Results

**Figure 1:**
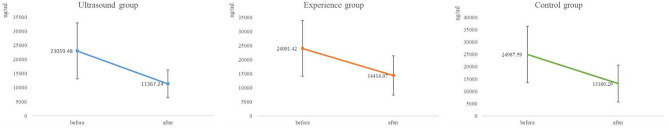
Changing trend of NT-proBNP among three groups.

**Figure 2:**
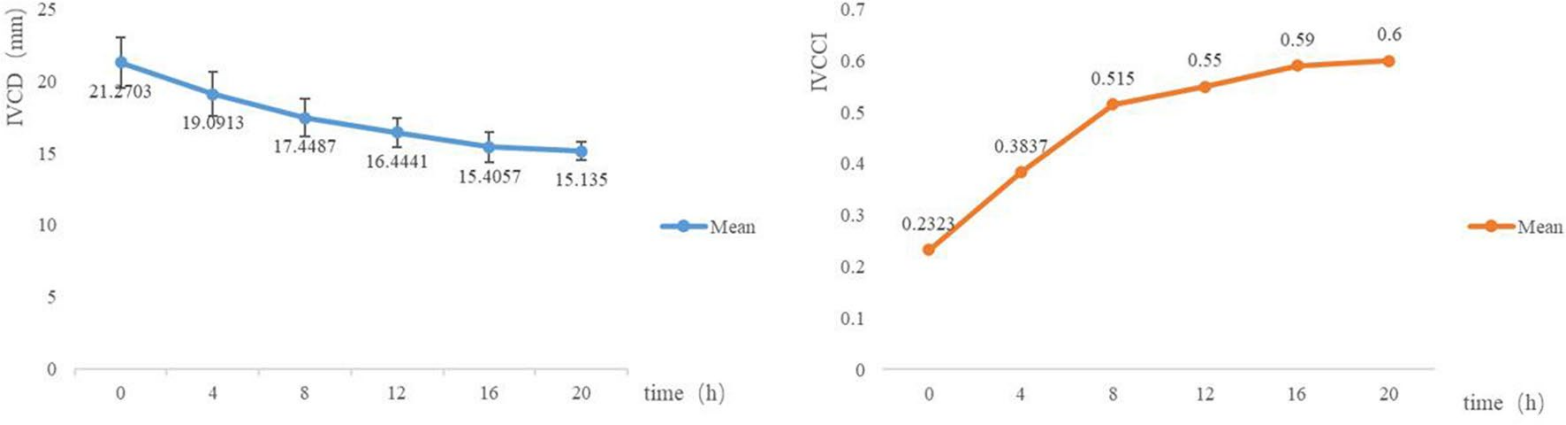
Changing trend in average IVCD and IVCCI values in the first CRRT among 30 patients in the ultrasound group after ICU admission.

The serum creatinine, potassium, and NT-proBNP levels among the three groups of patients decreased within 24 h after CRRT. The differences within the groups were statistically significant (P < 0.05), although there were no significant differences observed in pairwise comparisons among the three groups (P > 0.05) (Table 3). To more intuitively reflect the volume changes, we also plotted the changes in NT-proBNP, IVCD, and IVCCI (Figs. 1 and 2).

**Table 3:** Comparison of creatinine, potassium, and NT-proBNP levels among three groups before and 24 h after CRRT.

Group	Cases	Before			After		
		Creatinine	Potassium	NT-proBNP	Creatinine	Potassium	NT-proBNP
Ultrasound	30	817.08 ± 162.77	5.67 ± 0.64	23,059.48 ± 9964.45	410.97 ± 95.90^#^	4.08 ± 0.43^#^	11,367.24 ± 4921.83^#^
Experience	29	803.87 ± 140.48	5.59 ± 0.83	24,001.42 ± 9850.54	400.15 ± 87.14^#^	4.11 ± 0.31^#^	14,414.07 ± 6989.10^#^
Control	29	827.25 ± 151.29	5.66 ± 0.75	24,987.59 ± 11,492.37	422.60 ± 93.40^#^	4.14 ± 0.32^#^	13,160.29 ± 7501.93^#^
*T*		0.17	0.09	0.25	0.43	0.22	1.61
*P*		0.842	0.91	0.78	0.65	0.80	0.21

^#^Statistical significance compared with before CRRT, *P* < 0.05.

**Figure 3:**
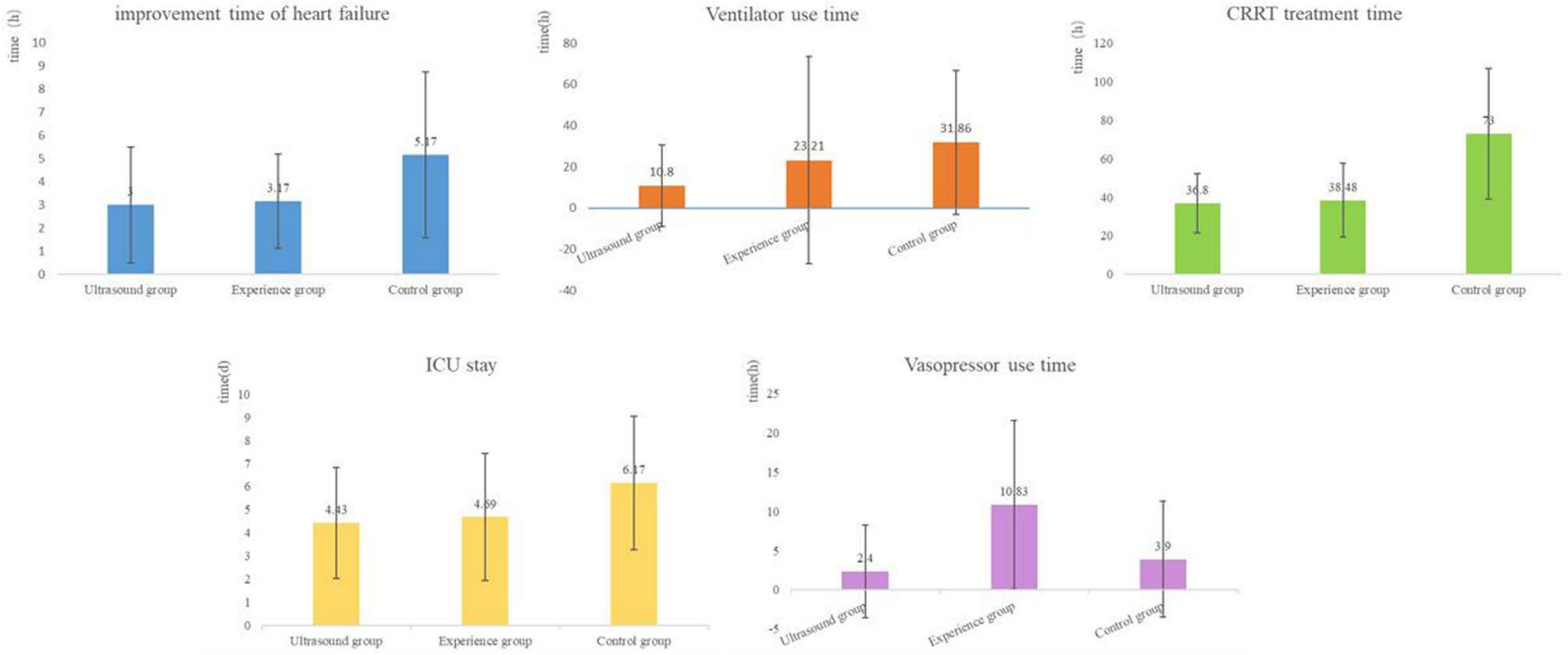
Comparison of observation indicators among three groups.

The time to improved heart failure, CRRT time, and ICU length of stay in the ultrasound and experience groups were significantly lower than those of the control group. The differences were statistically significant (P < 0.05), whereas there were no significant differences in the above indicators between the ultrasound and experience groups (P > 0.05) (Fig. 3).

The duration of ventilator use in both the ultrasound and experience groups was lower than that of the control group. The difference between the ultrasound and control groups was statistically significant (P < 0.05), whereas no significant difference was observed between the experience and control groups, or between the experience and ultrasound groups (P > 0.05).

The ultrasound and control groups used vasopressors for less time than the experience group, and the difference was statistically significant (P < 0.05), whereas the ultrasound and control groups showed no significant difference (P > 0.05) (Table 4).

**Table 4:** Comparison of clinical indicators among three groups.

Group	Cases	Time to improved heart failure symptoms (h)	CRRT time (h)	Ventilator use duration (h)	ICU length of stay (days)	Vasopressor use duration (h)
Ultrasound	30	3.00 ± 2.51	36.80 ± 15.27	10.80 ± 19.88	4.43 ± 2.40	2.40 ± 5.91*
Experience	29	3.17 ± 2.04	38.48 ± 19.09	23.21 ± 50.43	4.69 ± 2.75	10.83 ± 10.74
Control	29	5.17 ± 3.58^#,^*	73.00 ± 33.93^#,^*	31.86 ± 34.91^#^	6.17 ± 2.89^#,^*	3.90 ± 7.38*
*T*		5.50	21.05	2.41	3.57	8.72
*P*		*P* < 0.05	*P* < 0.05	0.10	*P* < 0.05	*P* < 0.05

^#^Statistical significance compared with the ultrasound group, *P* < 0.05.

*Statistical significance compared with the experience group, *P* < 0.05.

Adverse events occurred in 5 of 30 patients in the ultrasound group (5 cases of hypotension and 1 case of arrhythmia), 16 of 29 patients in the experience group (16 cases of hypotension, 4 cases of arrhythmia, and 1 case of delirium), and 16 of 29 patients in the control group (7 cases of hypotension, 8 cases of arrhythmia, and 6 cases of delirium). The incidence of adverse events in the ultrasound group was significantly lower than that in the experience and control groups, and the difference was statistically significant (P < 0.05). Conversely, the difference between the experience and control groups was not statistically significant (P > 0.05) (Table 5).

**Table 5:** Comparison of incidence of adverse events.

Group	Cases	Incidence of adverse events
Ultrasound	30	16.7%
Experience	29	55.2%^#^
Control	29	48.3%^#^
*x*^*2*^		10.43
*P*		*P* < 0.05

^#^Statistical significance compared with ultrasound group, *P* < 0.05.

## Discussion

Renal failure combined with acute heart failure involves a complex pathophysiological process. Metabolites and excessive fluid volume in the body cannot be discharged by the damaged kidneys; therefore. the accumulation of metabolites and fluid increases the heart load and can even result in acute heart failure^11^.

Renal failure and heart failure interact and aggravate each other, forming a vicious circle, eventually leading to sharp deterioration in heart and kidney function, which is a serious threat to patient safety ^12^. In clinical practice, CRRT is often used to replace the ability of the kidneys to remove excess fluid volume and metabolic products from the body to improve the patient’s condition^13^. However, the optimal method to achieve rapid and safe relief of heart failure symptoms remains unclear. Therefore, it is critical to correctly evaluate the patient’s blood volume status to facilitate CRRT dehydration adjustments.

Currently, the mainstream methods for assessing blood volume include the use of pulmonary artery catheters, pulse assessment (indicating continuous cardiac output), transoesophageal echocardiography, and bioimpedance^14–17^. These techniques have advantages, as well as many limitations. Many clinicians still prefer to assess the blood volume of patients using general empirical methods, such as estimating the patient’s dry weight, assessing for presence of pulmonary moist rales or lower extremity and facial oedema, and evaluating changes in vital signs. Although these approaches are simple and easy, they have poor reliability and cannot meet the requirements for rapid, dynamic, accurate, and non-invasive clinical evaluations.

In this study, we used ultrasonic and empirical methods to determine the blood volume of patients in the ultrasound and experience groups and compared the results with those of the control group. We found that serum creatinine, potassium, and NT-proBNP levels among all three groups decreased within 24 h of CRRT, and there were no significant differences in pairwise comparisons among the three groups, indicating that different blood volume assessment methods do not affect the serum creatinine and potassium clearance efficiency during the initial treatment, and no significant effect was observed on the NT-proBNP level.

We also found that the time to improved heart failure, CRRT time, and ICU length of stay in the ultrasound and experience groups were significantly shorter than those in the control group. Compared with the control group, the ultrasound group had significantly shorter ventilator use duration, and the difference was statistically significant. The above results show that, compared with the control group without fluid volume assessment, the ultrasound and experience groups had faster improvement in heart failure symptoms and shorter CRRT times and ICU hospitalisation durations.

Our study demonstrates that timely dynamic fluid volume assessment during CRRT has better clinical value in guiding dehydration adjustments in patients with renal failure combined with acute heart failure.

In the comparisons of vasopressor use and incidence of adverse events (i.e., hypotension, arrhythmia, delirium), we found that the duration of vasopressor use in the ultrasound and control groups was significantly lower than that in the experience group, and the incidence of adverse events (hypotension, arrhythmia, delirium) in the ultrasound group was significantly lower than that in the experience and control groups.

We considered several reasons for these results. First, the experiential method has a certain value in the assessment of patients with high-volume status, such as rapid improvement of heart failure symptoms, reduced CRRT time and length of ICU stay, whereas in patients with insufficient volume status, its accuracy remains uncertain. Heart rate and blood pressure will increase reflectively in patients with insufficient fluid volume, which may present as a pseudo-high-volume status leading to rapid dehydration during CRRT, thereby increasing the incidence of hypotension and duration of vasopressor use. Second, the patients in the control group had slow and uniform dehydration. Although the duration of vasopressor use was shorter, the remission of heart failure symptoms was slower, CRRT time significantly increased, length of ICU stay was prolonged, and incidence of adverse events such as arrhythmia and delirium increased. Third, the duration of ventilator use was significantly longer than the time to improved heart failure symptoms in all three groups, possibly because the patients’ oxygen content improved after mechanical ventilation. Moreover the heart failure symptoms improved significantly, although the patients’ blood volume remained in an overloaded state. Heart failure symptoms recurred if the ventilator was discontinued. Therefore, the ventilator duration time should be extended to ensure that the patient’s heart failure symptoms do not recur.

In contrast, the heart failure symptoms improved rapidly, and CRRT time, length of ICU hospitalisation, and ventilator use duration were significantly shortened in the ultrasound group. More importantly, the incidence of CRRT-related hypotension, vasopressor use duration, and incidence of adverse events was significantly reduced.

The primary limitation of our study is that it was a single-centre study with a small sample size. Hence, multicentre prospective studies with large sample sizes are warranted to validate our conclusions and provide a better basis for clinicians.

In conclusion, owing to the rapid progression of renal failure combined with acute heart failure, blood volume evaluation needs to be more intuitive and accurate. Ultrasonic dynamic monitoring of IVCD and IVCCI can provide accurate guidance for CRRT dehydration adjustment in patients with renal failure combined with acute heart failure. It can quickly relieve heart failure symptoms, reduce the incidence of adverse events and ICU treatment costs, and improve the patients' quality of life. Therefore ultrasonic dynamic monitoring of the IVCD and IVCCI has good social and economic benefits.

## Data Availability

The datasets used and/or analysed during the current study available from the corresponding author on request.
